# Isothiazolinones as Novel Candidate Insecticides for the Control of Hemipteran Insects

**DOI:** 10.3390/antibiotics10040436

**Published:** 2021-04-14

**Authors:** Wenze He, Lilong Pan, Wenhao Han, Xiaowei Wang

**Affiliations:** Ministry of Agriculture Key Laboratory of Molecular Biology of Crop Pathogens and Insects, Institute of Insect Sciences, Zhejiang University, Hangzhou 310058, China; 12016107@zju.edu.cn (W.H.); panlilong@zju.edu.cn (L.P.); wenhao_han@zju.edu.cn (W.H.)

**Keywords:** benzisothiazolinone, hemiptera, insecticide, isothiazolinone, Kathon, octylisothiazolinone

## Abstract

Hemipteran insects, such as whiteflies, aphids and planthoppers, resemble one of the most important pest groups threating food security. While many insecticides have been used to control these pests, many issues such as insecticide resistance have been found, highlighting the urgent need to develop novel insecticides. Here, we first observed that a commercial tetramycin solution was highly effective in killing whitefly. The major bioactive constituents were identified to be isothiazolinones, a group of biocides. We then tested the toxicity of several isothiazolinones to five hemipteran insects. The results show that Kathon, a widely used biocide against microorganisms, and its two constituents, chloromethylisothiazolinone (CMIT) and methylisothiazolinone (MIT), can cause considerable levels of mortality to whiteflies and aphids when applied at concentrations close to, or lower than, the upper limit of these chemicals permitted in cosmetic products. The results also indicate that two other isothiazolinones, benzisothiazolinone (BIT) and octylisothiazolinone (OIT) can cause considerable levels of mortality to whitefly and aphids but are less toxic than Kathon. Further, we show that Kathon marginally affects whitefly endosymbionts, suggesting its insecticidal activity is independent of its biocidal activity. These results suggest that some isothiazolinones are promising candidates for the development of a new class of insecticides for the control of hemipteran pests.

## 1. Introduction

As the global population will continue to grow to roughly nine billion by the middle of this century, sustaining crop production is vital, which entails, among others, efficient crop protection against insect pests [1,2]. Among insect pests, hemipterans such as whiteflies, aphids and planthoppers, are one of the most significant groups threating food security as they damage crops directly by feeding and indirectly by transmitting plant diseases [3,4]. Some species of the *Bemisia tabaci* whitefly complex are pests of many crops worldwide [5,6]. Whitefly feeding on crops may result in reduced plant vigor and physiological perturbation; more importantly, whiteflies can transmit many plant viruses belonging to the genera *Begomovirus* (*Geminiviridae*), *Crinivirus* (*Closteroviridae*), *Torradovirus* (*Secoviridae*), among others [7,8,9]. Aphids represent a serious challenge for the sustainable production of many important cereal and vegetable crops; they can remove plant sap, secret phytotoxic salivary components and transmit viruses such as potyviruses [10]. Likewise, planthoppers are economically important pests of many cereals such as rice; they feed directly or serve as vectors of pathogenic microorganisms including viruses, resulting in significant crop damage and yield losses [11].

While the importance of integrated pest management is being increasingly realized, the application of chemical pesticides remains necessary, and in many cases, the only crop protection measure in agricultural practices [1]. Although many compounds, such as organophosphates, carbamates, pyrethroids, and more recently, neonicotinoids are effective in many circumstances, to control of hemipteran pests, extensive applications of these chemicals have led to the emergence of resistance in these insects in addition to off-target effects on non-target organisms and the environment [11,12,13,14]. Based on this scenario, it is imperative to develop new classes of insecticides, preferably with novel modes of action. However, nowadays the identification and development of new insecticides is accomplished by only a limited number of companies, probably due to the fact that the development of a new insecticide is often very costly, in terms of both financial input and time [15]. Hence, identifying new chemicals with insecticidal properties from existing compounds, presently not used as insecticides, may contribute to the development of novel insecticides.

Tetramycin was obtained from the fermentation of *Streptomyces ahygroscopicus* subsp. Wuzhouensis. It exhibits significant antibiotic activity against a wide range of microorganisms such as bacteria and fungi [16]. In China, tetramycin has been widely used to treat fungal diseases in plants [17]. In an experiment to eliminate endosymbionts in whiteflies, we accidentally noticed that treatment of whiteflies with a commercial tetramycin solution resulted in high mortality within 48 h. Since tetramycin has no insecticidal activity, we postulated that some other components in the formulated tetramycin solution may kill whiteflies. We analyzed the tetramycin solution using Liquid chromatography-mass spectrometry (LC-MS) and surprisingly found that isothiazolinones were present in the solution and have insecticidal activity.

**Figure 1:**
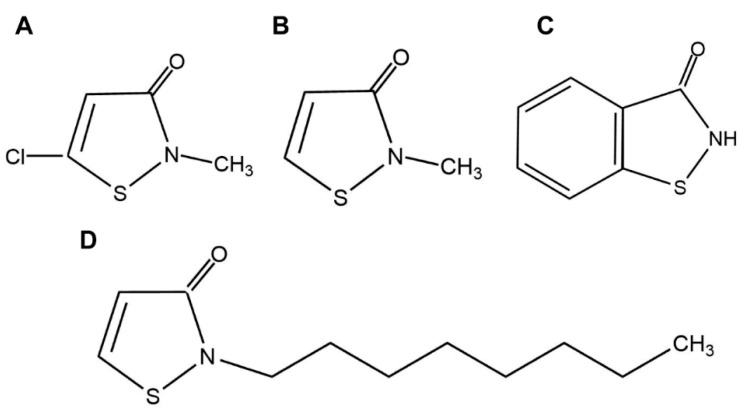
Chemical structure of CMIT (**A**), MIT (**B**), BIT (**C**) and OIT (**D**).

**Figure 2:**
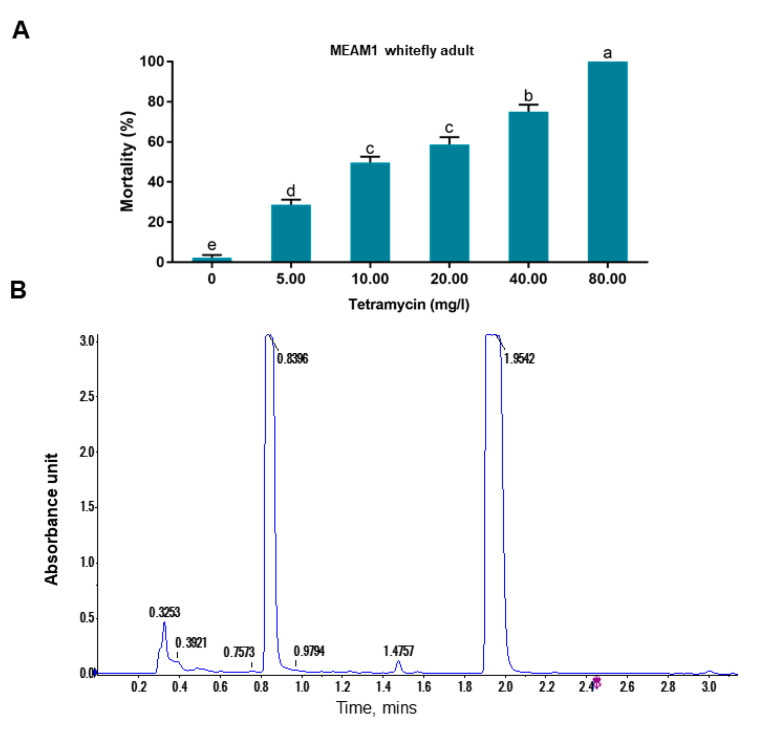
Effects of tetramycin treatment on whitefly survival and LC-MS analysis of the formulated tetramycin solution. Solutions of 15% sucrose, containing various concentration of formulated tetramycin solution were prepared and presented to whitefly orally by membrane feeding (**A**). LC-MS analysis profile of formulated tetramycin solution (**B**). Different letters above bars in A indicate significant difference (*p* < 0.05, one-way analysis of variance and Fisher’s LSD test). Peaks in B represent different chemicals detected in liquid chromatography.

Isothiazolinones are a class of broad spectrum microbicides that have been widely used to control the growth of microorganisms such as bacteria, fungi and yeast in cooling water systems and cosmetics, among others [18]. However, whether they can be used as insecticides for the control of agricultural pests, remains unexplored. We tested the most widely used isothiazolinone Kathon, which is a combination of 5-chloro-2-methyl-4-isothiazolin-3-one (Chloromethylisothiazolinone, CMIT) (Figure 1A) and 2-methyl-4-isothiazolin-3-one (Methylisothiazolinone, MIT) (Figure 1B) in an approximate 3:1 ratio. We assessed the toxicity of Kathon to five species of hemipteran insects including three species from the *B. tabaci* whitefly complex, the green peach aphid *Myzus persicae* and the rice brown planthopper *Nilaparvata lugens*. Further, we assessed the toxicity of another two widely used isothiazolinones 1,2-Benzisothiazolin-3-One (Benzisothiazolinone, BIT) (Figure 1C) and 2-N-octyl-4-isothiazolin-3-one (Octylisothiazolinone, OIT) (Figure 1D) to whitefly and the green peach aphid. Finally, we explored the effects of Kathon treatment on the density of endosymbionts in whitefly. Our results provide novel insights into the insecticidal properties of isothiazolinones, and highlight the potential of these compounds in the development of novel insecticides for the control of hemipteran pests.

## 2. Materials and Methods

### 2.1. Insects

Three species of whiteflies of the *B. tabaci* complex, namely Middle East-Asia Minor 1 (MEAM1), Mediterranean (MED) and Asia II 1, were tested. The mt*COI* GenBank accession codes were KM821540 for MEAM1, GQ371165 for MED and DQ309077 for Asia II 1. A laboratory culture of each of the three species of whiteflies was originally established from field samples, collected in Zhejiang, China in 2012 (MEAM1), 2009 (MED) and 2010 (Asia II 1). Whiteflies were maintained on cotton plants (*Gossypium hirsutum* cv. Zhemian1793) in climate-controlled chambers at 26 ± 2 °C, 60–80% relative humidity and a 14/10 h light/dark cycle without any contact with insecticides. Unless specified otherwise, adult whiteflies 1–7 days post emergence were used in the experiments.

A laboratory culture of the green peach aphid *M. persicae* was originally established from a field sample collected from field-grown tobacco plants (*Nicotiana tabacum*) on the Zijingang Campus of Zhejiang University, Hangzhou, China in 2018. The aphid culture was maintained on tobacco plants (*N. tabacum* cv. NC89) in a climate-controlled chamber at 23 ± 2 °C, 50–60% relative humidity and a 16/8 h light/dark cycle without any contact with insecticides.

Test insects of the rice brown planthopper *N. lugens* were provided by Professor Hai-Jun Xu (Institute of Insect Sciences, Zhejiang University) [19]. The culture of brown planthopper was collected in 2008 and maintained thereafter on rice seedlings (*Oryza sativa* cv. Xiushui 134) in a climate-controlled chamber at 26 ± 0.5 °C, 50–60% relative humidity and a 16/8 h light/dark cycle without any contact with insecticides.

### 2.2. Chemicals

Tetramycin (0.3% in water) was purchased from Liaoning Wkioc Bioengineering Co., Ltd. (Liaoning, China). Kathon (3% in glycol) and MIT (Figure 1B) (9.5% in water) were purchased from Sigma-Aldrich. CMIT (Figure 1A) (1.5% in water), BIT (Figure 1C) (85% in water) and OIT (Figure 1D) (100%) were purchased from Hubei Jusheng Technology Co., Ltd. (Hubei, China). Acetamiprid (20% in water) was purchased from Shandong Pharmaceutical Co., Ltd. (Shandong, China).

### 2.3. Analysis of Insecticidal Activity of Tetramycin and Kathon against Whiteflies via Oral Route

To assess the insecticidal activity of tetramycin solution against whitefly, 15% sucrose solutions containing 80, 40, 20, 10, 5 and 0 mg/L tetramycin were prepared. Similarly, 15% sucrose solutions containing 600.00, 300.00, 150.00 and 0 mg/L Kathon were prepared. Membrane feeding was conducted as mentioned before [20]. Five replicates were conducted for each concentration and 50 whiteflies were tested in each replicate.

### 2.4. Liquid Chromatography-Mass Spectrometry (LC-MS)

LC-MS was employed to identify the bioactive chemicals in tetramycin solution as reported before [21]. Waters Ultra Performance Liquid Chromatography (UPLC) and ACQUITY UPLC HSS SB-C18 column (1.7 μm, 2.1 × 100 mm) (Waters, MA, USA) was used in the chromatographic experiments. The mobile phases were 0.1% formic acid-water and 0.1% formic acid-acetonitrile. Sample injection volume, 3 μL; column oven temperature, 30 °C; flow rate, 0.4 mL/min; and UV detector was set at 254 nm. AB TripleTOF 5600plus System (AB SCIEX, Framingham, MA, USA) was employed for mass spectrometry. The optimal MS conditions: negative ion mode: Source voltage was −4.5 kV, and the source temperature was 550 °C. Positive ion mode: Source voltage was +5.5 kV, and the source temperature was 600 °C. Maximum allowed error was set to ±5 ppm. Decluttering potential (DP), 100 V; collision energy (CE), 10 V. For MS/MS acquisition mode, the parameters were the same except that the collision energy (CE) was set at 40 ± 20 V, ion release delay (IRD) at 67, ion release width (IRW) at 25. The IDA-based auto-MS2 was performed on the eight most intense metabolite ions in a cycle of full scan (1s). The scan range of *m*/*z* of precursor ion and product ion were set as 100–2000 Da and 50–2000 Da. The exact mass calibration was performed automatically before each analysis employing the Automated Calibration Delivery System. The data were analyzed using Peakview1.2 (AB SCIEX, Framingham, MA, USA).

### 2.5. Analysis of Insecticidal Activity of Kathon against Whiteflies via Spraying

To assess the insecticidal activity of Kathon against the adults of MEAM1 whitefly via spraying, the chemical was firstly diluted with water containing 1% surfactant (Tween-80) to six concentrations, namely 150.00, 75.00, 37.50, 18.75 and 9.38 mg/L. Water containing 1% surfactant (Tween-80) was used as negative control, and Acetamiprid (30.00 mg/L in water) was included as positive control. For MEAM1 nymphs, Asia II 1 and MED adults, 18.75 and 9.38 mg/L Kathon were used. For tests with whitefly adults, the test insects were confined on cotton leaves and then Kathon solutions were sprayed onto leaves. Specifically, in each replicate, approximately 100 whitefly adults were collected and released into a clip cage, which was covered with gauze and placed on the undersurface of a cotton leaf. Two hours later, live whiteflies were counted as the starting number of insects to be tested. The clip cage was then sprayed with a test solution using a hand-held sprayer as previously reported [22,23]. The gauze used allowed for efficient penetration of chemical solutions. For each of the four treatments of a whitefly species/stage, three replicates were conducted. Forty-eight hours later, the number of live and dead adult whiteflies were counted, and individuals were counted as dead when they were observed with a lack of discernible movement or obvious desiccation [24]. For the tests with MEAM1 whitefly nymphs, approximately 100 adult whiteflies were firstly collected and released into a clip cage placed on the undersurface of a cotton leaf. Fourteen days later, whitefly adults were removed, and the nymphs were then counted as the test insects (60–180 nymphs of all instars). Spray of Kathon solutions was then conducted as described above. The number of live nymphs was counted 48 h later, and dehydrated nymphs were considered dead.

### 2.6. Insecticidal Activity of CMIT and MIT against MEAM1 Whitefly

To assess the insecticidal activity of CMIT and MIT against MEAM1 whitefly adults, each of the two chemicals was diluted with water containing 1% surfactant (Tween-80) to two concentrations, namely 9.38 and 18.75 mg/L. Negative and positive controls were prepared as described above. Protocols of application of the test solutions and observation of mortality were conducted via spraying as above for Kathon. For each of the four treatments of CMIT or MIT, five replicates were conducted. 

### 2.7. Insecticidal Activity of Kathon against the Green Peach Aphid

Kathon solutions of various concentrations (18.75 and 9.38 mg/L), negative (water containing 1% Tween-80) and positive controls (30.00 mg/L Acetamiprid in water) were prepared, as described above. Leaves (30–50 cm^2^) of tobacco plants grown in greenhouses without any insecticide treatment were collected, soaked in Kathon solutions for 10 s and air-dried. Wingless aphid adults or nymphs were collected using a fine brush and placed onto the treated leaves, 15 individuals per leaf. The leaves were placed on agarose medium in 9 cm petri dishes to retain freshness. Live aphids were counted 48 h post transfer, and aphids that were completely immobile when touched upon or dehydrated were considered dead. For each of the four treatments for CMIT or MIT, three replicates were conducted.

### 2.8. Insecticidal Activity of Kathon against the Rice Brown Planthopper

A preliminary trial indicated that the rice brown planthopper was far less susceptible to Kathon than whiteflies or the green peach aphid. For the formal test, here, Kathon was diluted with water, containing the 1% surfactant (Tween-80) to two concentrations, namely 150.00 and 300.00 mg/L. Negative and positive controls were prepared, as described above. Rice plants that were grown in greenhouses without any insecticide treatment and at the booting stage were collected and washed thoroughly. Rice stems (about 10 cm in length) with roots were cut, air-dried, dipped in Kathon solutions in groups of three for 30 s and then air-dried again for 2 h. The rice stems were then covered with moistened cotton wool around the roots and placed into 500 mL plastic cups, one group per cup. Next, 20 third-instar nymphs or adults of the rice brown planthopper were transferred into each plastic cup using a fine brush. Live planthoppers were counted at 48 h post transfer, and planthoppers that were completely immobile when touched upon or dehydrated were considered dead. Three replicates were conducted for each of the four treatments for adults or nymphs.

### 2.9. Insecticidal Activity of BIT and OIT against the MEAM1 Whitefly and the Green Peach Aphid

By consulting the results of a preliminary trial, BIT and OIT were diluted with water containing 1% surfactant (Tween-80) to a final concentration of 150.00 mg/L. Negative and positive controls were prepared as described above. The mortality of MEAM1 whitefly adults or aphid adults was assessed as described above. Three replicates were conducted for each of the four treatments for the MEAM1 whitefly or the green peach aphid.

### 2.10. Analysis of Relative Endosymbiont Density Following Kathon Treatment

Kathon solutions of 4.69 and 9.38 mg/L were prepared and water containing 1% surfactant (Tween-80) was used as negative control. Whiteflies were collected and released into leaf-clip cages, and then Kathon solutions were applied by spraying as mentioned above. Forty-eight hours later, whiteflies were collected in groups of 15 and then subjected to DNA extraction and analysis of endosymbionts as reported before [25]. Briefly, lysis buffer (5 mmol/L of Tris-HCl (pH 8.0), 0.5 mmol/L EDTA, 0.5% Nonidet P-40 and 1 mg/mL of proteinase K) was used for whitefly DNA extraction. Endosymbionts were quantified by quantitative real-time PCR (qPCR) with SYBR Premix Ex TaqTM (Takara, Japan) and Bio-Rad CFX96 Real- Time System (Bio-Rad, Hercules, CA, USA) with primers listed in Shan et al. (2019)

### 2.11. Statistical Analysis

For the analysis of whitefly mortality, all percentage data were arcsine square root transformed prior to statistical analysis, and back-transformed for presentation. Relative endosymbiont density was calculated as normalized to whitefly *actin*. For the comparisons of whitefly mortality and endosymbiont density among different treatments, one-way analysis of variance, followed by Fisher’s least significance divergence (LSD) test was used. The differences between treatments were considered significant when *p* < 0.05. All data are presented as mean ± standard error of mean. All statistical analyses were performed using SPSS 20.0.

## 3. Results

### 3.1. Effects of Tetramycin Treatment on the Survival of MEAM1 Whitefly via Oral Route and Identification of Bioactive Constituents

We first explored the effects of tetramycin treatment on the survival of MEAM1 whitefly (Figure 2A). The mortality of whitefly increased with increasing concentrations of tetramycin, reaching 100.0% when the concentration was 80 mg/L. Since no insecticidal activity of tetramycin has been reported, but has been used in the field for many years, we speculated some other components in the formulated tetramycin solution may be responsible for killing whiteflies. We used LC-MS and identified two chemicals in the formulated tetramycin solution we used (Figure 2B), and followed MS analysis identified these chemicals as 2- methyl-4-isothiazolin-3-one (Figure S1) and 2-methyl-4-isothiazolin-3-one hydrochloride (Figure S2). Both chemicals are members of isothiazolinones, a group of broad-spectrum biocides.

### 3.2. Effects of Kathon on the Survival of Three Species of Whiteflies

**Figure 3:**
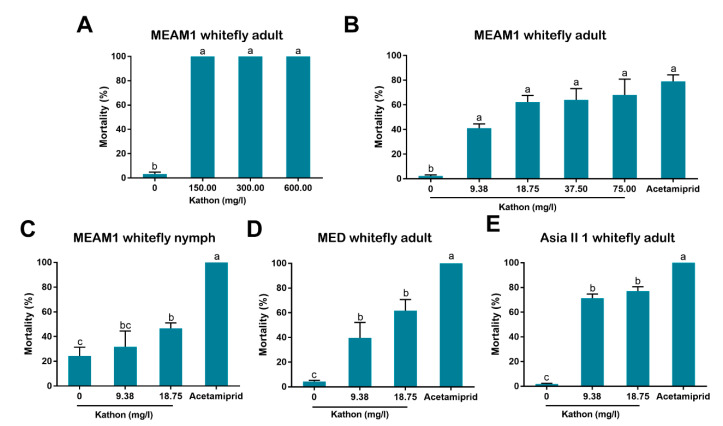
Effects of Kathon on the survival of three species of whiteflies of the *Bemisia tabaci* complex via oral route and spraying. Membrane feeding was conducted to determine the effects of Kathon on the survival of MEAM1 adults via oral route (**A**). Spraying application was performed for MEAM1 adults (**B**), MEAM1 nymphs (**C**), MED adults (**D**) and Asia II 1 adults (**E**). For spraying, Kathon was diluted with water containing 1% surfactant (Tween-80) to two concentrations. Acetamiprid (30 mg/L) was used as positive control. The solutions were sprayed onto plants, and the mortality of whitefly adults and nymphs was assessed at 48 h post treatment. For each of the four treatments of a given whitefly species/stage, three replicates were conducted with each containing approximately 100 adults or 50–200 nymphs. Different letters above bars indicate significant difference (*p* < 0.05, one-way analysis of variance and Fisher’s LSD test).

We then tested the efficacy of Kathon, the most widely used isothiazoline with a combination of CMIT and MIT in an approximate 3:1 ratio, against the adults of MEAM1 whitefly via oral route, and found that Kathon killed 100% of whiteflies when concentrations were 600.00, 300.00 and 150.00 mg/L (Figure 3A). Next, a spraying method was employed for whitefly bioassay and lower concentrations were used. Acetamiprid were included as a positive control. When MEAM1 adults were treated with Kathon at the concentrations of 75.00, 37.50, 18.75 and 9.38 mg/L, considerable levels of mortality were observed, though not as high as that observed with application of acetamiprid (Figure 3B). Likewise, treatment of MEAM1 whitefly nymphs with 18.75 mg/L Kathon, resulted in a considerable level of mortality, and no significant mortality was observed with the treatment of 9.38 mg/L Kathon (Figure 3C). Considerable levels of mortality were also observed in adults of both MED (Figure 3D) and Asia II 1 (Figure 3E) when they were treated with 9.38 and 18.75 mg/L Kathon.

### 3.3. Effects of CMIT and MIT on the Survival of MEAM1 Whitefly Adults

**Figure 4:**
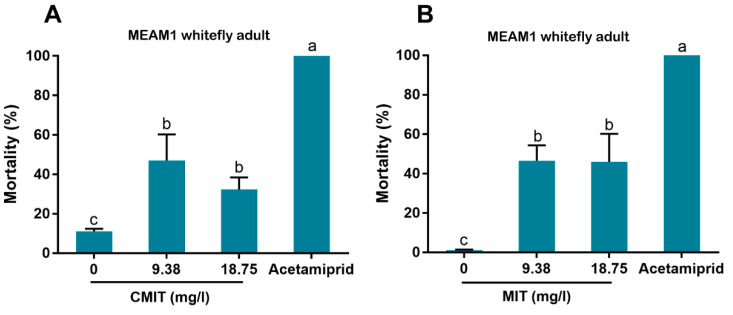
Effects of CMIT (**A**) and MIT (**B**) on the survival of MEAM1 adults. CMIT and MIT were diluted with water containing 1% surfactant (Tween-80) to two concentrations, respectively. Acetamiprid: 30 mg/L. The solutions were sprayed onto the plants, and the mortality of whitefly adults and nymphs was assessed at 48 h post treatment. For each of the four treatments for CMIT or MIT, five replicates were conducted on groups of approximately 100 adults or 50–200 nymphs. Different letters above bars indicate significant difference (*p* < 0.05, one-way analysis of variance and Fisher’s LSD test).

To examine which components of Kathon have the insecticidal activity, we treated MEAM1 whitefly adults with CMIT or MIT individually at the concentrations of 9.38 and 18.75 mg/L. Considerable levels of mortality were observed, though significantly lower than those observed following application of acetamiprid (Figure 4A,B).

### 3.4. Effects of Kathon on the Survival of the Green Peach Aphid

**Figure 5:**
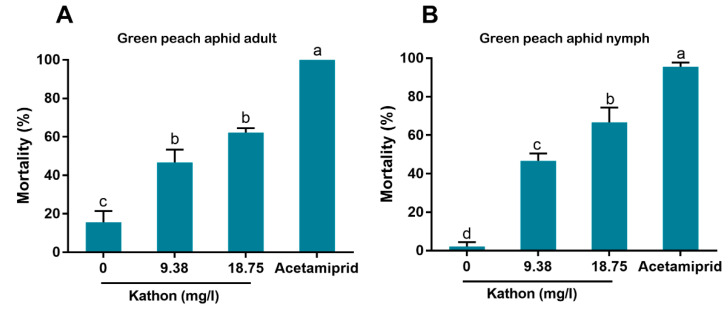
Effects of Kathon on the survival of the green peach aphid *Myzus persicae*: adult (**A**) and nymph (**B**). Acetamiprid (30 mg/L) was used as a positive control. The solutions were applied using dipping, and the mortality of aphid adults and nymphs was assessed at 48 h post treatment. For each of the four treatments of adult or nymph, three replicates were conducted on groups of 15 adults or nymphs. Different letters above bars indicate significant difference (*p* < 0.05, one-way analysis of variance and Fisher’s LSD test).

When aphid adults were treated with Kathon at concentrations of 9.38 and 18.75 mg/L, the mortalities were 46.7% and 62.2% (Figure 5A). When aphid nymphs were treated with Kathon at 9.38 and 18.75 mg/L, the mortalities were 46.7% and 66.7% (Figure 5B).

### 3.5. Effects of Kathon on the Survival of the Rice Brown Planthopper

**Figure 6:**
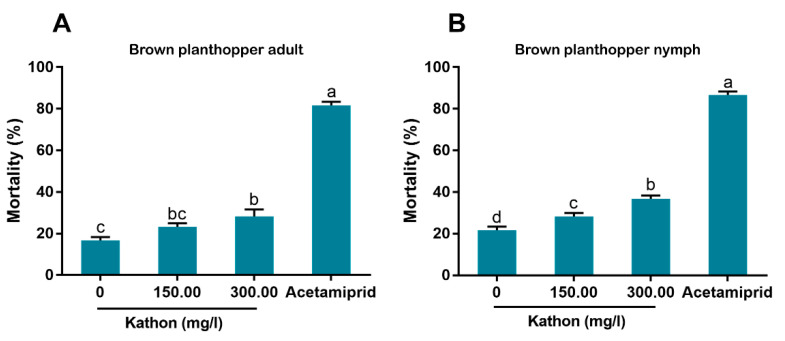
Effects of Kathon on the survival of rice brown planthopper *Nilaparvata lugens*: adult (**A**) and nymph (**B**). Acetamiprid (30 mg/L) was used as a positive control. The solutions were applied to rice-stems by dipping, and mortality of brown planthopper adults and nymphs was assessed at 48 h post treatment. For each of the four treatments of adults or nymphs, three replicates were conducted on groups of 20 individuals. Different letters above bars indicate significant difference (*p* < 0.05, one-way analysis of variance and Fisher’s LSD test).

As adults of the rice planthopper were treated with Kathon at a concentration of 300 mg/L, a moderate level of mortality was observed, though significantly lower than that observed following application of acetamiprid. Whereas, no significant mortality was observed when treated with Kathon at the concentration of 150.00 mg/L (Figure 6A). Similarly, when nymphs of the rice brown planthopper were treated with Kathon at the concentrations of 150.00 and 300.00 mg/L, moderate levels of mortality were observed and the level of mortality increased at the higher concentration, though still significantly lower than that observed following application of acetamiprid (Figure 6B).

### 3.6. Efficacy of BIT and OIT against Adults of the MEAM1 Whitefly and the Green Peach Aphid

**Figure 7:**
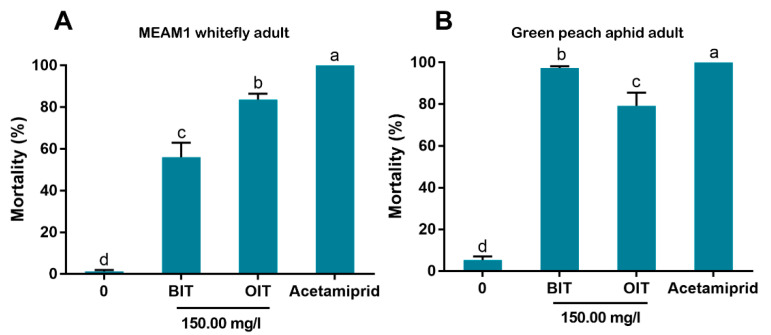
Effects of BIT and OIT on the survival of MEAM1 whitefly adults (**A**) and adults of the green peach aphid (**B**). Acetamiprid (30 mg/L) was used as a positive control. Whitefly and aphid mortality was recorded at 48 h post treatment. For each of the four treatments of the whitefly or aphid, three replicates were conducted. Each replicate of whitefly comprised approximately 100 adults and each replicate of aphid comprised 15 aphid adults. Different letters above bars indicate significant difference (*p* < 0.05, one-way analysis of variance and Fisher’s LSD test).

When adults of the MEAM1 whitefly were treated with BIT or OIT at a concentration of 150 mg/L, considerable levels of mortality were observed, the mortality caused by OIT was significantly higher than that caused by BIT, but significantly lower than that observed following application of acetamiprid (Figure 7A). When adults of the green peach aphis were treated with BIT or OIT, high levels of mortality were observed, and the mortality caused by by BIT approached 100%, which was observed following application of acetamiprid (Figure 7B).

### 3.7. Effects of Kathon Treatment on the Density of Endosymbionts in Whitefly

**Figure 8:**
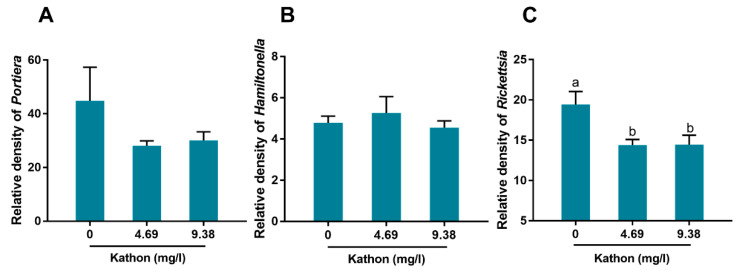
Effects of Kathon treatment on the density of endosymbionts in whitefly. Whiteflies were treatment with Kathon via spraying, and 48 h later the density of *Portiera* (**A**), *Hamiltonella* (**B**) and *Rickettsia* (**C**) were analyzed. Different letters above bars indicate significant difference (*p* < 0.05, one-way analysis of variance and Fisher’s LSD test).

To explore whether Kathon treatment would affect the density of endosymbionts in whitefly, three endosymbionts *Portiera*, *Hamiltonella* and *Rickettsia* were analyzed. For the primary endosymbiont *Portiera*, Kathon treatment did not significantly change its density in whitefly (Figure 8A). Likewise, the density of secondary endosymbionts *Hamiltonella* did not significantly change upon Kathon treatment (Figure 8B). However, a significant decrease of *Rickettsia* density was observed; specifically, 9.38 and 4.69 mg/L Kathon treatment decreased *Rickettsia* density by 25.7%, and 26.0%, respectively (Figure 8C).

## 4. Discussion

In this study, we accidentally observed that a commercial formulated tetramycin solution can effectively kill whiteflies (Figure 2). In the process of tracing the insecticidal components in the tetramycin solution, we identified two isothiazoline biocides that exhibited insecticidal activity against whitefly. As isothiazoline is widely used as preservatives in many solutions [26], we presumed that it may have been added to the tetramycin solution during production. We next tested the effects of the most widely used isothiazoline biocide Kathon and its two constituents CMIT and MIT on the survival of five species of hemipteran insects.

In our bioassay, we first used high concentrations (up to 600 mg/L) and then used lower concentrations by sequentially diluting the higher ones. Finally we chose 9.38 and 18.75 mg/L for bioassay as the European Union regulations on cosmetic products states that the upper limit of isothiazolinones such as Kathon in leave-on and rinse-off cosmetics is 15 mg/L [27]. Our results indicate that Kathon and its two constituents can cause considerable levels of mortality to three species of whiteflies from the *B. tabaci* complex and the green peach aphid when applied at the concentration close to, or lower than, the upper limit of these chemicals, permitted by the European Union regulations on cosmetic products (Figure 3, Figure 4 and Figure 5). Interestingly, Kathon seemed to be more potent against whitefly, suggesting a synergistic effect between CMIT and MIT (Figure 3 and Figure 4). Additionally, brown planthoppers seem to be less susceptible to Kathon than the other insects (Figure 6). The low susceptibility of brown planthoppers may be due to many reasons, for example the insect may degrade Kathon efficiently, among other. Nevertheless, further investigations are warranted to verify these hypotheses. The results also show that the two other isothiazolinones BIT and OIT were less toxic to the MEAM1 whitefly and the green peach aphid than Kathon and its two constituents (Figure 7). Overall, the data indicate that some isothiazolines can exert considerable levels of mortality on some hemipteran insects, such as whiteflies and aphids.

In recent decades, neonicotinoid insecticides have been widely used in the control of phloem-feeding insect pests, such as whiteflies, aphids and planthoppers [13,14]. The extensive application of neonicotinoids in the field has seen rapid development of insect resistance to insecticides of this class, such as imidacloprid and thiamethoxam [13,28]. Rotated application of insecticides with different modes of action has proven to be an effective strategy for managing resistance [28]. To implement this strategy, the development of new insecticides, with novel modes of action that are benign to the environment, is necessary. Isothiazolones inhibit microbial growth and metabolism by inhibiting critical physiological processes, such as respiration and energy generation [29,30,31]. Further, isothiazolones may react with many thiol-containing compounds such as cysteine, thereby forming disulfide derivatives and in turn promoting a series of reactions that impair key cellular functions [29,31,32]. While mechanisms underlying the toxicity of isothiazolones to hemipteran insects are unknown, disruption of critical cellular processes and interaction with thiol-containing compounds may both be possible. Nevertheless, the mechanisms of action are likely to differ significantly from those of neonicotinoids that act selectively on the insect central nerve system as agonists of the postsynaptic nicotinic acetylcholine receptors [14]. With regard to the mechanism of action, here we observed that isothiazolone treatment marginally affected the density of endosymbionts in whitefly. However, elimination of endosymbionts in hemipteran insects such as whitefly does not affect the survival of treated insects [25,33,34]. Hence, the microbicidal property of isothiazolones may contribute little, if any, to the insecticidal activity of these compounds.

Isothiazolinones are effective, fast-acting chemicals for the inhibition of microbial growth and metabolism and biofilm development. They are widely used for many industrial purposes and as a preservative in cosmetics including lipsticks and shampoo; under ambient conditions, isothiazolinones are quite stable as their estimated half-life is six months [26]. While isothiazolinones, such as Kathon have been reported to cause skin sensitization in humans and may induce apoptosis in human keratinocytes [35]. A review of case studies indicates that they are safe to human when the concentration is below 15 mg/L [18]. When plants were sprayed with isothiazolinones at the concentrations used in our experiments (up to 150 mg/L), the plants did not suffer any appreciable sign of toxicity (data not shown). These results suggest that use of isothiazolinones as insecticides at a concentration lower than 15mg/L may have limited risks to humans and crops. However, as broad spectrum biocides, isothiazolinones may significantly impact organisms other than insect pests. Therefore, investigations on the effects of isothiazolinones to non-target organisms are necessary in evaluating their potential use as insecticides.

Taken together, this study has identified the insecticidal properties of commonly used biocidal isothiazolinones including Kathon, BIT and OIT. Our findings indicate that isothiazolinones are promising candidates for the development of a new class of insecticides for the control of hemipteran pests.

## Data Availability

Data in this study is available upon request.

## Supplementary Materials

The following are available online at https://www.mdpi.com/article/10.3390/antibiotics10040436/s1, Figure S1: MS ion chromatograms of the substance peaked at 0.8396 min in LC analysis and Figure S2: MS ion chromatograms of the substance peaked at 1.9542 min in LC analysis.
