# Supplementary material for: Isothiazolinones as Novel Candidate Insecticides for the Control of Hemipteran Insects
Source: Antibiotics (Basel). 2021 Apr 14;10(4):436. doi: 10.3390/antibiotics10040436 (PMC8069810; doi:10.3390/antibiotics10040436)
Supplement: Supplementary file 1 [file antibiotics-10-00436-s001.pdf]

antibiotics

Article

Isothiazolinones as Novel Candidate Insecticides for the Control of Hemipteran Insects

Wen-Ze He, Li-Long Pan, Wen-Hao Han and Xiao-Wei Wang *

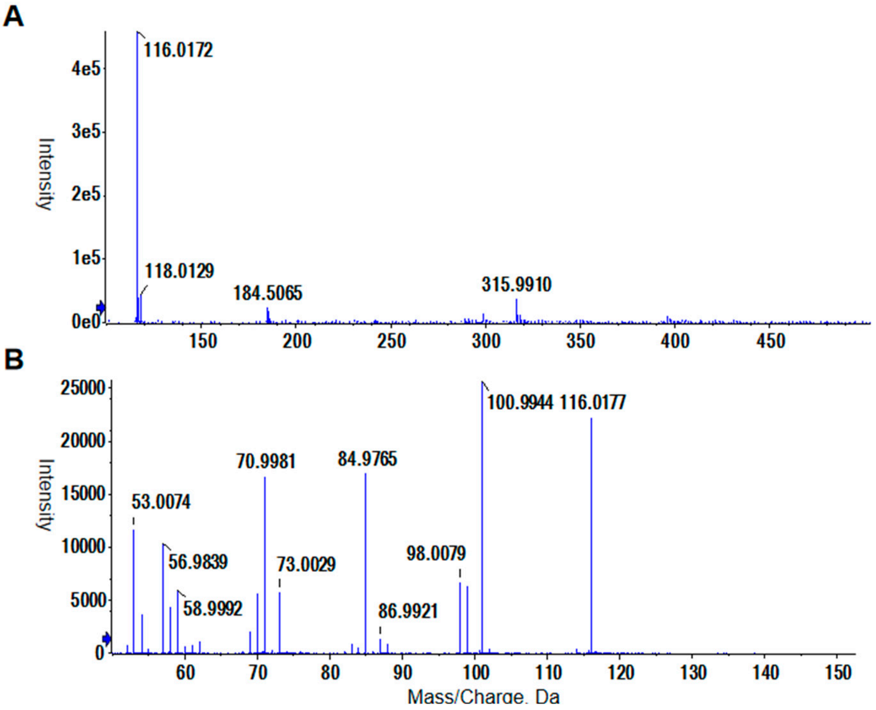

Figure S1. MS ion chromatograms of the substance peaked at 0.8396 min in LC analysis. Analysis of ion chromatograms revealed the chemical is 2-methyl-4-isothiazolin-3-one.

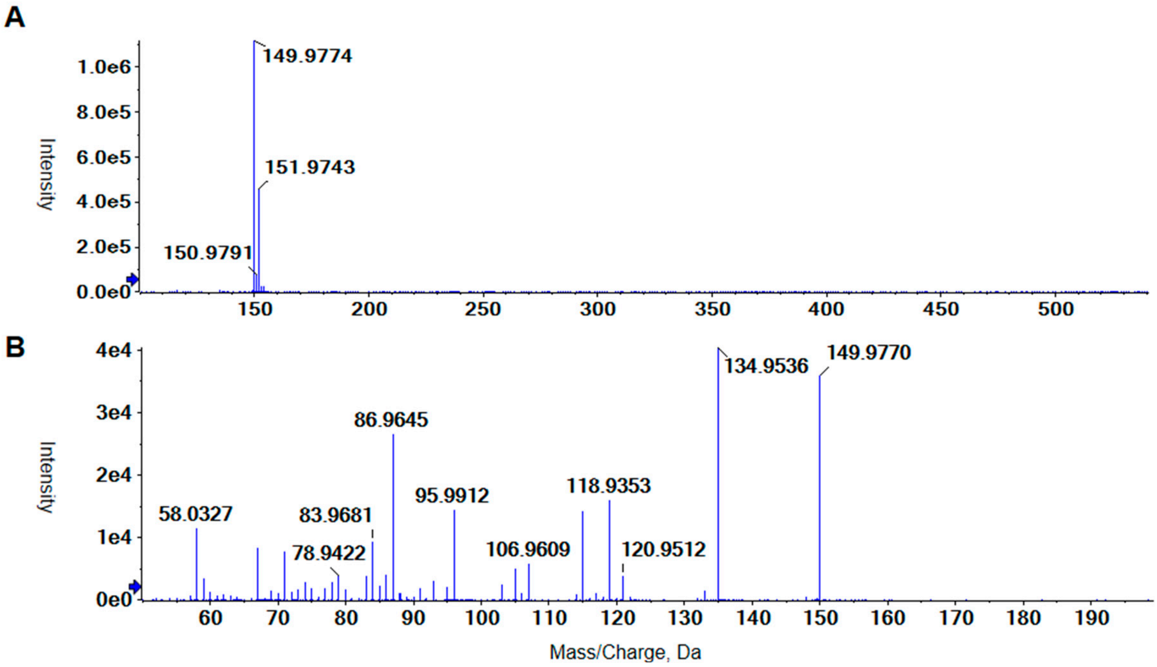

Figure S2. MS ion chromatograms of the substance peaked at 1.9542 min in LC analysis. Analysis of ion chromatograms revealed the chemical is 2-methyl-4-isothiazolin-3-one hydrochloride.
